# Strain-specific probiotic properties of lactic acid bacteria and their interference with human intestinal pathogens invasion

**DOI:** 10.1186/s13099-017-0162-4

**Published:** 2017-03-06

**Authors:** Raffaella Campana, Saskia van Hemert, Wally Baffone

**Affiliations:** 1 Division of Toxicological, Hygiene and Environmental Sciences, Department of Biomolecular Science, University of Urbino “Carlo Bo”, Urbino, Italy; 2 Winclove Probiotics, Amsterdam, The Netherlands

**Keywords:** Lactic acid bacteria, Probiotic properties, Interference, Gut pathogens

## Abstract

**Background:**

One of the working mechanisms of probiotic bacteria is their ability to compete with pathogens. To define the probiotic properties of seven Lactic Acid Bacteria (LAB) strains, we tested them for survival in simulated gastro-intestinal conditions, antimicrobial activities, co-aggregative abilities, and interferences studies against five human intestinal pathogens (*Salmonella enteritidis* ATCC 13076, *Listeria monocytogenes* ATCC 7644, *Escherichia coli* O157: H7 ATCC 35150, *Cronobacter sakazakii* ATCC 29544 and *Campylobacter jejuni* ATCC 33291).

**Results:**

The LAB strains were able to survive the stomach simulated conditions, and varied in their abilities to survive the small intestinal-simulated conditions. The strains showed antibiotic susceptibility profiles with values equal or below the breakpoints set by the European Food and Safety Authority. The LAB cell-free cultures supernatants showed antimicrobial activities, with inhibition zones ranging from 10.0 to 17.2 mm. All the LAB strains showed moderate auto-aggregation abilities while the greatest co-aggregation abilities were observed for *Bifidobacterium bifidum* W23, *Lactobacillus plantarum* W21 and *Lactobacillus rhamnosus* W71. The individual LAB strains showed strain-specific abilities to reduce the invasion of intestinal pathogens in an interference model with Caco-2 cells. Increased invasion inhibition was found when different combinations of LAB strains were used in the interference tests.

**Conclusion:**

The LAB strains examined in this study may protect the intestinal epithelium through a series of barriers (antimicrobial activity, co-aggregation with pathogens, adherence) and interference mechanisms. Consequently, these LAB strains may be considered candidates for prophylactic use to prevent intestinal infections.

**Electronic supplementary material:**

The online version of this article (doi:10.1186/s13099-017-0162-4) contains supplementary material, which is available to authorized users.

## Background

The infectious diseases caused by food-borne pathogens are a serious public health threat as reported by Centers for Disease Control and Prevention (CDC) and Foodborne Diseases Active Surveillance Network (FoodNet) [1]. Food spoilage-inducing bacterial pathogens such as *Escherichia coli* O157: H7 (EHEC), *Salmonella*, *Listeria monocytogenes*, and *Campylobacter* cause numerous illnesses and deaths, and huge economical loss [2]. Diarrhea, often caused by these pathogens, is the second leading cause of death in children under 5 years old and kills over 2 million people overall, per year [3]. Whereas most of the deaths occur in developing countries, also in developed countries a lot of foodborne and waterborne infectious illnesses occur, with up to 1 in 6 of Americans affected yearly [4]. Treatment is mainly done by oral rehydration solution and anti-motility agents like loperamide are used widely [4]. Commonly used synthetic antibiotics are efficient in limiting the growth of food-borne pathogens, but a growing numbers of antibiotic-resistance among those human pathogens have been documented [5].

Probiotic bacteria represent a potential alternative in the prevention and control of food-borne infections, since they have proven effectiveness in reversing the pathogenicity of food-borne pathogens. Probiotics are defined as live microorganisms that confer a health benefit on the host when administered in adequate amounts [6, 7]. Strains of lactic acid bacteria (LAB) belonging to the genera *Lactobacillus* and *Bifidobacterium* are commonly used as probiotics [8]. The mechanisms underlying the activity of LAB strains against bacterial pathogens appear to be multifactorial and include the production of hydrogen peroxide, lactic acid, bacteriocin-like molecules, stimulation of the immune system, and modulation of intestinal microbiota [9–11]. Moreover, LAB can prevent the adhesion of pathogens by competing for the binding sites on the intestinal epithelial cells and consequently, reduce the colonization, thereby preventing the onset of infection [12–14]. In order to extent beneficial effects, probiotics need to achieve in the intestine an adequate biomass through growth, biofilm formation or aggregation and, consequently, the ability to aggregate is a desirable property for probiotics. In addition, micro-organisms with the ability to co-aggregate with other bacteria, such as pathogens, may have an advantage over the non-co-aggregating bacteria that are easily removed from the intestinal gut [15].

Dietary intervention through food or food supplements containing live microbes with the aforementioned properties could be a possible step to improve the intestinal health status of people and to prevent infectious diarrhoea caused by food-borne pathogens. However, it is well-known that different bacterial strains of the same genus and species may exert completely different effects on the host [16]. Therefore, the specific properties of individual strains should be well-defined and the effect on health of each strain should be demonstrated in a case-by-case manner.

For these reasons, the potential probiotic properties of different LAB strains against *Salmonella enteritidis* ATCC 13076, *L. monocytogenes* ATCC 7644, *E. coli* O157: H7 ATCC 35150, *Cronobacter sakazakii* ATCC 29544 and *Campylobacter jejuni* ATCC 33291 were determined in this study. The experimental design was subdivided in two distinct phases in order to: (i) determine the strain-specific probiotic properties of the different LAB, evaluating their antimicrobial activity as well as auto- and co-aggregation properties; (ii) study the interference of selected LAB strains and their combinations against the invasion ability of human intestinal pathogens.

## Methods

### Bacterial strains and culture conditions

Seven strains of lactic acid bacteria (from the probiotic formulation WincloveTravel), kindly provided by Winclove Probiotics (The Netherlands), were used in this study: *Bifidobacterium bifidum* W23 (DSM 26331), *Lactobacillus salivarius* W24 (DSM 26403), *Lactobacillus acidophilus* W37 (DSM 26412), *Lactobacillus casei* W56 (DSM 26388), *Lactococcus lactis* W58 (DSM 26390), *Lactobacillus plantarum* W21 (DSM 26401) and *Lactobacillus rhamnosus* W71 (DSM 26396). These seven lactic acid strains are deposited at the DSMZ culture collection. They have been identified based on the highest match of a partial DNA sequence of the small subunit (16S) ribosomal RNA gene of the tested strain with the sequences of different LAB species, in the database of the Ribosomal Database Project II (RDP release 9.56), or based on rep-PCR fingerprint profile similarity to a reference culture that was identified as such based on the highest match of a partial DNA sequence of the small subunit (16S) Ribosomal RNA gene of the tested strain with the sequence of different LAB species in the database of the Ribosomal Database Project II (RDP release 9.56).

All the probiotic strains were grown on Man Rogosa Sharpe agar (MRS, Oxoid, Milan, Italy) for 24–48 h at 37 °C under microaerophilic conditions (5% O_2_; 10% CO_2_, 85% N_2_); *B. bifidum* W23 was grown in MRS with the addition of 0.05% cysteine in the same culture conditions. For interference studies, five reference human intestinal pathogens *S. enteritidis* ATCC 13076, *L. monocytogenes* ATCC 7644, *E. coli* O157: H7 ATCC 35150, *C. sakazakii* ATCC 29544 and *C. jejuni* ATCC 33291, were included. All pathogenic strains were grown in Tryptic Soy agar (TSA, Oxoid) at 37 °C for 24 h, while *C. jejuni* ATCC 33291 was grown on Columbia Agar Base (Oxoid) with 5% Laked Horse Blood (Oxoid) and *Campylobacter* Growth supplement (Oxoid) at 37 °C for 48 h under microaerophilic conditions. All lactic acid bacteria and pathogenic strains were stored at −80 °C in Nutrient Broth No. 2 (Oxoid) with 20% of glycerol.

#### pH and bile tolerance tests

The survival of the lactic acid bacteria to simulated gastro-intestinal (GI) tract conditions was investigated as described previously [17]. Briefly, each lyophilized strain (2 g, 10^9^ CFU/gr) was rehydrated in 100 ml of demineralized water for 15 min at room temperature. The rest of the experiment was performed at 37 °C in a water bath. The stomach was simulated by adding 1 ml of porcine pepsin solution (7 mg/ml porcine gastric mucosa p7000, Sigma) and decreasing the pH in four steps of 15 min to 4.8, 4.5, 3.5 and 2.5. After 75 min, the entry into the proximal duodenum was mimicked by increasing the pH to 6.5 by adding 0.1 N NaOH and, after 90 min, 10 ml of porcine bile extract solution (80 mg/ml of bile extract, Sigma) and 2 ml of porcine pancreatin solution (50 mg/ml pancreatin, Sigma) were added. After 3 h, bile salts were deactivated by adding 11.5 mM of calcium chloride. The pH was maintained at 6.5 until 6 h, which was the end of the experiment. Samples for the total cell count analysis were taken at the different time points, diluted in phosphate buffered saline and plated in several dilutions on MRS agar plates. Plates were incubated for 48–72 h at 37 °C and thereafter the colony forming units (CFU/ml) were counted. The experiments have been performed in triplicate.

### Antibiotic susceptibility testing

The antibiotic resistance for all the LAB strains used in this study was checked by the broth micro dilutions method. The bacteria were tested for resistance against ampicillin, vancomycin, gentamycin, kanamycin, streptomycin, erythromycin, clindamycin, tetracycline and chloramphenicol (VetMIC Lact-1 and 2 micro-dilution plates). The bacteria were plated onto MRS agar plates and grown for 24–48 h. A single colony of each strain was diluted in saline solution (McFarland 0.5) and distributed over 96 wells microtiter plates with LAB susceptibility medium [18] and different concentrations of antibiotics with a final bacterial load of 10^5^ CFU/ml. The plates were incubated at 37 °C for 24 h. The Minimal Inhibitory Concentration (MIC) was determined as the lowest concentration of a given antibiotic at which no growth of the tested organism was observed and compared with the breakpoints set by the European Safety and Food Authority [19].

### Preparation of pathogen strain inoculums

Pathogen strains represented by *S. enteritidis* ATCC 13076, *L. monocytogenes* ATCC 7644, *E. coli* O157: H7 ATCC 35150, *C. sakazakii* ATCC 29544 were grown in Tryptic Soy Broth (TSB) (Oxoid) at 37 °C for 24 h, while *C. jejuni* ATCC 33291 was grown in Mueller–Hinton Broth (MHB) (Oxoid) supplemented with 5% of Fetal Calf Serum (FCS) (Sigma) with gentle shaking (120 rpm) at 37 °C for 48 h under microaerophilic conditions. At the end of the incubation period, for each experiment, the bacterial cultures were centrifuged at 3500 rpm for 15 min, resuspended in the adequate culture media and adjusted to a turbidity of about 10^8^ CFU/ml by spectrophotometer reader using OD_660_ for *C. jejuni* ATCC 33291 and OD_610_ for all the other intestinal pathogens [20, 21].

### Antimicrobial activity of LAB “cell-free cultures supernatants”

For the “cell-free cultures supernatant” (CFCS) extraction, the lactic acid bacteria were inoculated into 100 ml of MRS broth at 37 °C for 48 h under microaerophilic conditions. The obtained cultures were centrifuged at 12,000 rpm for 15 min at 4 °C and the supernatants (CFCSs), adjusted to pH 6.5, were filtered with pore size 0.22 µm membranes and stored at −20 °C until use.

The antibacterial properties of the CFCSs was determined using the agar well diffusion method (AWDM). Briefly, the pathogenic strains were grown overnight in TSB at 37 °C and then 500 µl of each culture (10^7^ CFU/ml) were added to 25 ml of Nutrient Agar. The plates were let solidify at room temperature for 20 min; subsequently, on their surface, 6 mm holes were aseptically created. Finally, each well was filled with 50 µl of the different CFCSs and the plates were incubated at 37 °C for 24 h. After incubation, the zones of growth inhibition around each well, considered index of antimicrobial activity of the CFCSs, were measured and registered. Each experiment was performed in triplicate.

#### Aggregative abilities of LAB strains

Auto-aggregation and co-aggregation abilities of each LAB strain were evaluated. For auto-aggregation ability, LAB strains were grown in MRS broth at 37 °C for 24 h under microaerophilic conditions, as described above. Then, the bacterial cultures were centrifuged at 3500 rpm for 10 min and the bacteria were resuspended in 10 ml of PBS to approximately 10^8^ CFU/ml (OD_550_ 0.2–0.3). Each suspension was vortexed for 10 s and incubated for 6 h at room temperature. At each hour, 1 ml of the upper part of each suspension was withdrawn to measure the absorbance at 600 nm. The percentage of auto-aggregation was then calculated according to the following formula:

$${\text{auto-aggregation }}\left( \% \right) = 1- \left( {{\text{A}}_{\text{t}} /{\text{A}}_{0} } \right) \times { 1}00$$

where A_t_ is the absorbance at different time points and A_0_ the initial one.

For co-aggregation abilities, 2-ml aliquots of pairs of bacterial suspensions (probiotic and pathogen) were vortexed for 10 s. Samples containing 4-ml aliquots of a single bacterial suspension were used as control. Each suspension was vortexed for 10 s and incubated for 6 h at room temperature. At each hour, 1 ml of the upper part of each suspension was withdrawn to measure the absorbance as described above. The co-aggregation percentages were finally calculated as follow:

$$\% {\text{Coaggregation = }}\frac{{\left( {{{\left( {{\text{A}}_{x} + {\text{A}}_{y} } \right)} /2}} \right){ - {\rm A}}\left( {x + y} \right)}}{{{{{\text{A}}_{x} + {\text{A}}_{y} } /2}}} \times 100$$

where A_x_ and A_y_ are the individual aggregation properties of the lactobacilli and the pathogen, and A(x + y) is the combined aggregation of the lactobacilli and the pathogen. All the experiments were performed in duplicate.

#### Cell cultures

Caco-2 cells, human colon carcinoma cells, were grown routinely in 25 cm^2^ flasks containing approximately 6 ml of D-MEM (Sigma) supplemented with 10% Fetal Calf Serum (FCS) (Sigma), 1% of non-essential amino acids (Sigma) and 1% of antibiotics (penicillin and streptomycin) (Sigma) at 37 °C with 5% CO_2_. For all the experiments, Caco-2 cells were treated with trypsin, seeded at a ratio of 2 × 10^4^ cells/ml in 6-well plates and used as differentiated cells after 15 days in culture. Before the assays, the cell monolayers were washed twice with phosphate buffered saline (PBS) pH 7.2

### Adhesive properties of LAB strains on Caco-2 cells

The adhesive properties of LAB strains were evaluated on Caco-2 monolayers prepared as described above. Briefly, a loopful of each LAB strain was transferred into a sterile glass tube containing 10 ml of MRS broth; for *B. bifidum* W23 was used MRS broth with 0.05% of cystein. All tubes were then incubated at 37 °C for 24 h under microaerophilic conditions. At the end of incubation, the bacterial cultures were centrifuged at 3500 rpm for 10 min and resuspended in D-MEM with 1% FCS. The bacterial density of each culture was adjusted to OD_600_ of 0.9–1 corresponding to about 10^8^ CFU/ml. These suspensions were co-incubated with Caco-2 monolayers for 1 h at 37 °C in 5% CO_2_. After the incubation period, supernatants were discarded and the monolayers were softly washed twice with phosphate saline buffer (PBS) to remove the non-attached bacteria. The monolayers were finally trypsinized to release the eukaryotic cells and adhered bacteria; after appropriate serial dilutions in physiological saline solution, the number of adhered bacteria was enumerated on MRS agar after incubation at 37 °C for 24 h under microerophilic conditions. Results were expressed as the percentage of bacteria adhered with respect to the amount of bacteria added (% CFU bacteria adhered/CFU bacteria added) [15].

### Invasion inhibition by interference studies with selected LAB strains and their combinations

Interference assays were carried out using *B. bifidum* W23, *L. salivarius* W24 and *L. rhamnosus* W71; each selected on the basis of their adhesive and aggregative abilities. Exclusion and competition tests were used as infection schemes on Caco-2 cell monolayers with specific time of infection for each pathogen: 2 h for *S. enteritidis* ATCC 13076 and *L. monocytogenes* ATCC 7644, 3 h for *E. coli* O157: H7 ATCC 35150 and *C. sakazakii* ATCC 29544, 4 h for *C. jejuni* ATCC 33291. The bacterial suspensions of LAB and pathogen strains were prepared as described above.

For the exclusion test, Caco-2 monolayers were infected with 1 ml of each LAB suspension for 1 h and then washed with PBS to remove non-adherent bacteria; at this point, 1 ml of each pathogenic suspension was added to cells monolayers for the appropriate incubation time at 37 °C with 5% of CO_2_. At the end of incubation, cells were washed 3–5 times with PBS, treated with D-MEM gentamicin solution (250 µg/ml) for 2 h and lysed with Triton X-100 (0.5% in PBS). Finally, cell lysates were serially diluted in physiological saline solution, plated on the adequate agar and incubated in the appropriate culture condition for the CFU/ml enumeration.

For the competition test, cells were exposed to a mixed suspension (1:1) of each pathogen with each LAB strain. After the appropriate incubation times at 37 °C with 5% of CO_2_, monolayers were washed 3–5 times with PBS, treated with D-MEM gentamicin solution (250 µg/ml), washed with PBS, and lysed with Triton X-100 (0.5% in PBS). The lysates were then serially-diluted in saline, and plated on the adequate agar and incubated in the culture condition for CFU/ml enumeration.

Subsequently, for each pathogen, the exclusion and competition exposure schemes were performed using the four following LAB strains combinations:

i. *B. bifidum* W23 + *L. salivarius* W24
ii. *B. bifidum* W23 + *L. rhamnosus* W71
iii. *L. salivarius* W24 + *L. rhamnosus* W71
iv. *B. bifidum* W23 + *L. salivarius* W24 + *L. rhamnosus* W71

The interference studies were performed as described above with the difference that Caco-2 cells were infected with pathogens and each LAB combination. Infection on Caco-2 cells, gentamicin killing protection assay, cellular lysis and viable counts were carried out as previously described.

All the interference assays were performed in duplicate.

### Statistical analysis

Statistical analysis was performed using Prism 5.0 (GraphPad Software, Inc., La Jolla, USA). The conditions necessary to perform parametric tests were checked before conducting the analysis, otherwise non-parametric tests were utilized. The level of significance was considered α = 0.05.

## Results

### Strain-specific probiotic properties

**Fig. 1:**
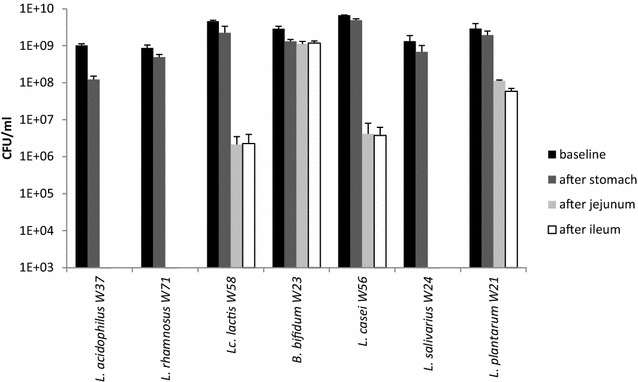
In vitro GI survival data of LAB strains. Each lyophilized strain (2 g, 10^9^ CFU/gr) was rehydrated in 100 ml of demineralized water for 15 min at room temperature (baseline). The rest of the experiment was performed at 37 °C. The stomach was simulated by adding 1 ml of porcine pepsine solution and decreasing the pH in four steps of 15 min to 4.8, 4.5, 3.5 and 2.5. After 75 min (stomach), the entry into the proximal duodenum was mimicked by increasing the pH to 6.5 by adding 0.1 N NaOH and, after 90 min, 10 ml of porcine bile extract solution and 2 ml of porcine pancreatin solution were added. After 3 h (duodenum), bile salts were deactivated by adding 11.5 mM of calcium chloride. The pH was maintained at 6.5 until 6 h (ileum) which was the end of the experiment. The experiments have been performed in triplicate

The in vitro GI survival data for the seven LAB strains are shown in Fig. 1. All the strains showed good survival to the simulated stomach conditions. In particular, *L. acidophilus* W37 showed a reduction of approximately one log CFU/ml, while the other strains had a much lower reduction of CFU/ml. The ability to survive to the simulated conditions of the small intestinal differed between the strains. In detail, *L. acidophilus* W37, *L. rhamnosus* W71, and *L. salivarius* W24 were unable to survive in this in vitro GI model, while *L. casei* W56 and *L. lactis* W58 showed a reduction of 3 log CFU/ml, and *L. plantarum* W21 of 1.5 log CFU/ml. Finally, *B. bifidum* W23 showed only a twofold reduction of CFU/ml value at the end of the experiment (Fig. 1).

The minimal inhibitory concentrations of nine different antibiotics for the seven LAB strains are summarized in Table 1; all values are equal or below the breakpoints set by the European Food and Safety Authority.

**Table 1:** Minimum inhibitory concentrations (MIC, mg/l) of the tested LAB strains for nine different antibiotics

	Amp	Van	Gen	Kan	Strep	Ery	Clin	Tetra	Chlo
*B. bifidum* W23	0.06 [2]	1 [2]	64 [64]	n.r.	32 [128]	0.25 [1]	0.12 [1]	2 [8]	2 [4]
*L. acidophilus* W37	0.094 [1]	0.5 [2]	1 [16]	32 [64]	3 [16]	0.016 [1]	0.094 [1]	0.38 [4]	1.5 [4]
*L. casei* W56	0.5 [4]	n.r.	4 [32]	64 [64]	32 [64]	0.25 [1]	0.12 [1]	2 [4]	8 [4]
*L. plantarum* W21	0.5 [2]	n.r.	8 [16]	64 [64]	n.r.	0.5 [1]	2 [2]	32 [32]	4 [8]
*L. rhamnosus* W71	4 [4]	n.r.	2 [16]	32 [64]	8 [32]	0.12 [1]	0.5 [1]	1 [8]	4 [4]
*L. salivarius* W24	1 [4]	n.r.	2 [16]	64 [64]	32 [64]	0.25 [1]	0.25 [1]	4 [8]	4 [4]
*L. lactis* W58	0.25 [2]	0.25 [4]	2 [32]	8 [64]	16 [32]	0.12 [1]	0.12 [1]	1 [4]	4 [8]

In square brackets are indicated the microbial breakpoint according to the European Food and Safety Authority [19]. Strains with MICs higher that the breakpoint are considered resistant

*Amp* ampicillin, *Van* vancomycin, *Gen* gentamycin, *Kan* kanamycin, *Strep* streptomycin, *Ery* erythromycin, *Clin* clindamycin, *Tetra* tetracycline, *Chlo* chloramphenicol, *n.r.* not required

### Antimicrobial activity of CFCSs against food-borne pathogens

The antimicrobial effect of CFCSs extracted from each LAB strain against selected food-borne pathogens are summarized in Table 2. The CFCSs tested in this experiment were able to inhibit the growth of intestinal pathogens with a variable degree of antibacterial activity. In fact, the CFCSs of *L. lactis* W58, *L. plantarum* W21, and *L. rhamnosus* W71 showed a wide antimicrobial activity against all the food-borne pathogens included in this study, whilst the others CFCSs have demonstrated a limited or absent antimicrobial activity.

**Table 2:** Antimicrobial activity of the cell-free supernatants (CFCSs) produced by the different LAB strains toward human intestinal pathogens strains performed by agar well diffusion method

CFCSs	Inhibition zone (mm ± sd)				
	*S. enteritidis* ATCC 13076	*L. monocytogenes* ATCC 7644	*E. coli* O157: H7 ATCC 35150	*C. sakazakii* ATCC 29544	*C. jejuni* ATCC 33291
*B. bifidum* W23	–	10.1 ± 0.25	11.0 ± 0.35	12.1 ± 0.25	12.1 ± 0.32
*L. salivarius* W24	10.1 ± 0.32	–	–	10.2 ± 0.11	10.1 ± 0.28
*L. acidophilus* W37	–	11.1 ± 0.12	–	–	12.1 ± 0.36
*L. casei* W56	–	11.1 ± 0.51	–	10.2 ± 0.21	17.1 ± 0.21
*L. lactis* W58	11.0 ± 0.15	11.0 ± 0.27	12.0 ± 0.28	11.1 ± 0.15	10.5 ± 0.51
*L. plantarum* W21	10.1 ± 0.31	14.1 ± 0.34	10.0 ± 0.20	10.1 ± 0.28	13.5 ± 0.52
*L. rhamnosus* W71	10.1 ± 0.24	12.1 ± 0.31	11.0 ± 0.15	10.2 ± 0.25	17.2 ± 0.21
Positive control	16.3 ± 0.53 (Gen)	19.3 ± 0.63 (W)	15.6 ± 0.64 (Gen)	16.3 ± 0.52 (Strep)	32 ± 0.51 (Gen)

*Gen* gentamicin 10 µg, *W* trimethoprim 5 µg, *Strep* streptomycin 10 µg, – no visible growth inhibition

Specifically, the greatest zones of growth inhibition, 17.2 ± 0.21 and 17.1 ± 0.21 mm, were reached by the CFCS of *L. rhamnosus* W71 and *L. casei* W56 toward *C. jejuni* ATCC 33291, the microorganism resulted more sensitive to all the examined CFCSs. The activity of CFCSs against the others four food-borne pathogens showed lower zones of growth inhibition.

### Aggregation abilities and adhesiveness of LAB strains

The auto- and co-aggregation abilities of the LAB strains are summarized in Table 3. After 6 h of incubation, the highest percentages of aggregation were seen for *B. bifidum* W23 and *L. rhamnosus* W71 (21.37 and 21.08% respectively). All the LAB demonstrated auto-aggregation ability higher than those showed by intestinal pathogens, whose percentage values ranging from a minimum of 10.30% for *E. coli* O157: H7 ATCC 35150 to a maximum of 12.90% for *S. enteritidis* ATCC 13076. Regarding the co-aggregation abilities, the LAB strains that showed the strongest co-aggregation after 6 h of incubation were *B. bifidum* W23, *L. plantarum* W21 and *L. rhamnosus* W71. Specifically, *B. bifidum* W23 showed the highest co-aggregation ability with *C. jejuni* ATCC 33291 (18.14%), *L. plantarum* W21 with *S. enteritidis* ATCC 13076 (16.79%), and *L. rhamnosus* W71 with *E. coli* O157: H7 ATCC 35150 (17.41%). All the probiotic strains were able to co-aggregate with *C. jejuni* ATCC 33291, except *L. acidophilus* W37 and *L. lactis* W58 (4.33 and 3.42%).

**Table 3:** Percentages of auto- and co-aggregation abilities of the different LAB strains with intestinal pathogens

	Auto-aggregation	Co-aggregation with pathogens				
		*S. enteritidis* ATCC 13076	*L. monocytogenes* ATCC 7644	*E. coli* O157: H7 ATCC 35150	*C. sakazakii* ATCC 51329	*C. jejuni* ATCC 33291
*B. bifidum* W23	21.37 (±1.12)	14.86 (±0.28)	11.96 (±0.20)	10.27 (±0.31)	10.07 (±0.28)	18.14 (±1.02)
*L. salivarius* W24	19.33 (±1.81)	6.82 (±0.20)	8.33 (±0.12)	10.31 (±0.21)	7.06 (±0.22)	15.84 (±0.32)
*L. acidophilus* W37	15.90 (±1.20)	14.2 (±0.19)	8.35 (±0.11)	3.85 (±0.01)	9.52 (±0.19)	4.33 (±0.02)
*L. casei* W56	15.92 (±1.72)	6.34 (±0.11)	5.14 (±0.15)	5.64 (±0.14)	5.34 (±0.11)	13.94 (±0.32)
*L. lactis* W58	15.50 (±1.21)	13.94 (±0.31)	7.13 (±0.13)	9.32 (±0.21)	4.54 (±0.31)	3.42 (±0.28)
*L. plantarum* W21	15.03 (±1.61)	16.79 (±0.35)	14.62 (±0.31)	12.01 (±0.24)	4.72 (±0.34)	13.00 (±0.21)
*L. rhamnosus* W71	21.08 (±1.83)	15.93 (±0.24)	13.73 (±0.22)	17.4 (±0.22)	12.51 (±0.26)	11.00 (±0.23)

Data were obtained after 6 h of incubation at room temperature. Data are expressed as mean ± SD

**Fig. 2:**
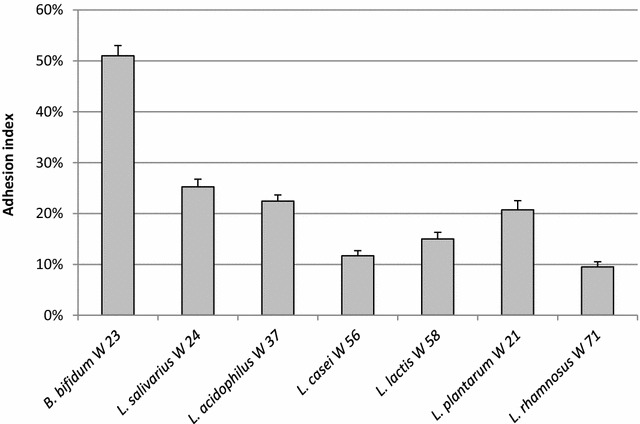
Adhesion ability of the different LAB strains to the human Caco-2 cell monolayers. Caco-2 monolayers were incubated for 1 h with a LAB strain. Thereafter the supernatants were discarded and the monolayers were washed to remove the non-attached bacteria. Then the monolayers were trypsinized to release the eukaryotic cells and adhered bacteria and the bacteria were plated on MRS plates. Results are expressed as the percentage of bacteria adhered with respect to the amount of bacteria added (% CFU bacteria adhered/CFU bacteria added). The experiments have been performed in triplicate

The adhesion abilities of each LAB strain on Caco-2 cell monolayers are represented in Fig. 2. In general, the examined LAB presented good adhesion ability to intestinal cells with strain-specific characteristics. More specifically, *B. bifidum* W23 showed the highest adhesion index (% CFU bacteria adhered/CFU bacteria added) of 51%, while the others strains evidenced adhesion indexes ranging from 25% for *L. salivarius* W24 to 9.5% for *L. rhamnosus* W71.

### Interference of LAB strains on intestinal pathogens invasion ability

The capacity of *B. bifidum* W23, *L. salivarius* W24, and *L. rhamnosus* W71 to inhibit the intestinal food-borne pathogens invasion on Caco-2 cells, selected on the basis of their adhesion index and aggregative abilities, was determined using exclusion and competition tests. The interference studies against each intestinal pathogen were performed using LAB strains singularly and four different combinations of LAB strains as described in “Methods” section.

**Fig. 3:**
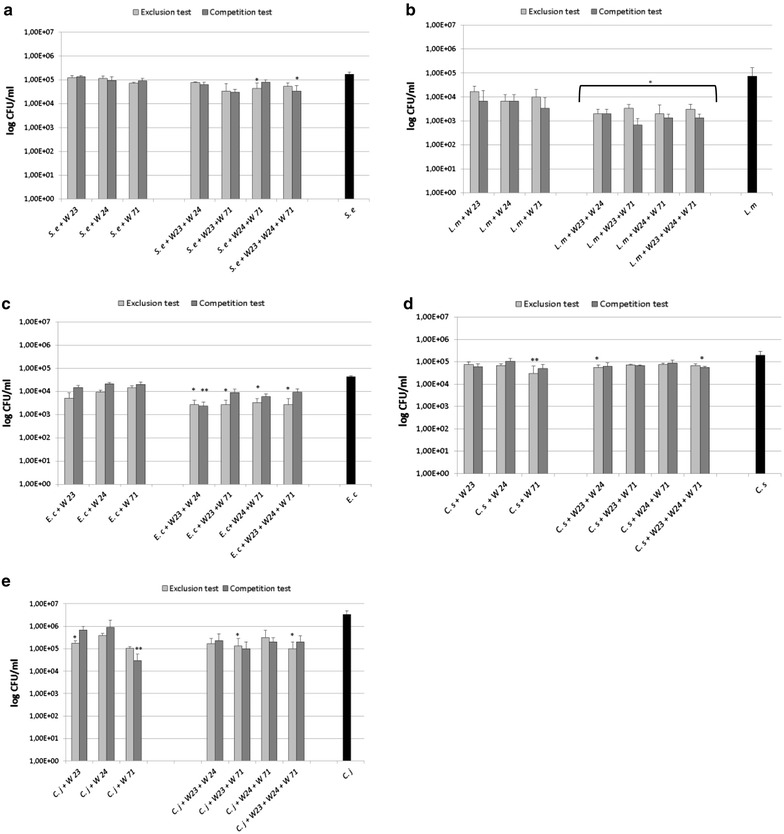
Invasion inhibition of human intestinal pathogens by single LAB strains and their four combinations. **a** *S. enteritidis* ATCC 13076, **b** *L. monocytogenes* ATCC 7644, **c** *E. coli* O157: H7 ATCC 35150, **d** *C. sakazakii* ATCC 29544, **e** *C. jejuni* ATCC 33291. *Asterisks* represented values statistically significant (p < 0.05) in comparison to the control group (Kruskal–Wallis non parametric test)

As a general trend, each tested LAB strain was able to inhibit the invasiveness of *S. enteritidis* ATCC 13076, *L. monocytogenes* ATCC 7644, *E. coli* O157: H7 ATCC 35150, *C. sakazakii* ATCC 29544 and *C. jejuni* ATCC 33291 with a variable degree dependent on the bacterial species (Fig. 3). Each single LAB strain showed weak ability to reduce the invasion of *S. enteritidis* ATCC 13076 on Caco-2 cells. The LAB combinations provoked a higher decrease of internalized *S. enteritidis* ATCC 13076 in both the interference tests (Fig. 3a).

The three single LAB strains demonstrated good interference activity against the invasion ability of *L. monocytogenes* ATCC 7644 (Fig. 3b), with a remarkable decrease of recovered internalized bacteria after gentamicin protection assay on Caco-2 cell monolayers. Statistically significant (p value <0.05) reductions of *L. monocytogenes* ATCC 7644 numbers were registered in the interference tests with all the four LAB combinations.

The tested LAB strains were also able inhibit the invasion ability of *E. coli* O157: H7 ATCC 35150 (Fig. 3c). As observed for the others tested intestinal pathogens, the four LAB combinations induced a more remarkable decrease of the internalized *E. coli* O157: H7 ATCC 35150 in comparison with the individual strains. The single LAB strains were able to weakly interfere with Caco-2 invasion ability of *C. sakazakii* ATCC 29544 (Fig. 3d), and 4.48 log CFU/ml, a statistically significant decrease of the recovered internalized bacteria, was registered only in the exclusion test performed with *L. rhamnosus* W71, compared to 5.30 log CFU/ml of the control internalized *C. sakazakii* ATCC 29544. In the interference studies carried out with the four LAB combinations, the most considerable decrease of the internalized *C. sakazakii* ATCC 29544 (4.75 log CFU/ml) was obtained in the exclusion tests with the combination formed by *B. bifidum* W23 and *L. salivarius* W24 and in the competition test with that formed by *B. bifidum* W23, *L. salivarius* W24 and *L. rhamnosus* W71.

Finally, regarding *C. jejuni* ATCC 33291, the obtained results indicated that each single LAB strains inhibited the invasion ability of *C. jejuni* ATCC 33291 (Fig. 3e). In detail, statistically significant reductions of the internalized *C. jejuni* ATCC 33291, compared to the control ones (6.52 log CFU/ml), were registered in the exclusion test with *B. bifidum* W23 (5.24 log CFU/ml) or *L. rhamnosus* W71 (5.01 log CFU/ml), while the lowest values of internalized *C. jejuni* ATCC 33291 was evidenced in the competition test with *L. rhamnosus* W71 (4.48 log CFU/ml). The four LAB combinations were also able to considerably reduce the number of internalized *C. jejuni* ATCC 33291.

The findings for all pathogens and all tested LAB strains and combination are summarized in Table 4.

**Table 4:** Invasion inhibition of single LAB strains and their combinations against human intestinal pathogens

Strains and interference test	Single LAB strains			LAB combinations			
	W23	W24	W71	W23 + W24	W23 + W71	W24 + W71	W23 + W24 + W71
*S. enteritidis* ATCC 13076							
Exclusion (%)	27.92	31.82	57.14	55.19	80.52	74.68	68.83
Competition (%)	20.13	45.45	47.40	62.99	82.47	53.25	80.52
*L. monocytogenes* ATCC 7644							
Exclusion (%)	76.94	90.78	86.16	97.23	95.39	97.23	95.85
Competition (%)	90.78	90.78	95.39	97.23	99.08	98.16	98.16
*E. coli* O157:H7 ATCC 35150							
Exclusion (%)	88.11	77.95	66.14	93.70	93.70	92.13	93.70
Competition (%)	65.35	49.61	52.76	94.49	78.74	85.83	77.95
*C. sakazakii* ATCC 29544							
Exclusion (%)	56.19	61.90	82.86	71.67	63.33	61.67	66.67
Competition (%)	65.71	23.81	52.38	68.33	66.67	56.67	71.67
*C. jejuni* ATCC 33291							
Exclusion (%)	94.80	88.30	96.90	95.00	96.00	90.70	97.00
Competition (%)	80.00	73.00	99.10	93.00	97.00	94.00	94.00

## Discussion

### Probiotic characterization of each LAB strain

The ability to survive an in vitro GI model varied between the tested LAB strains. Strain specific tolerance to stomach acidity and/or GI survival has been shown before [17, 22, 23]. Surprisingly, the main decrease in viable was found in the simulated conditions of small intestinal, and not under the acid stomach conditions. In general most LAB appear to possess a natural ability to survive pancreatin [24, 25]. In contrast pancreatin tolerance, bile tolerance is strain-specific [26], so this might cause the observed differences is survival. In vivo, the amount of bile shows high variability to time, along the length of the GI tract and amongst individuals, and the conditions in our in vitro model might not adequately reflect the in vivo situation, where food matrices might help survival of the bacteria.

Antibiotic resistance and transferability of antibiotic resistance genes from probiotic strains to commensal microbiota in the gut are important components of the safety of bacteria used as probiotics [27]. No antibiotic resistance was found in these commercial available LAB strains for the EFSA panel of antibiotics, whereas this has been found in some other studies [28]. These latter results highlights the importance of antimicrobial susceptibility testing as part of the safety analysis of potential probiotic strains.

In the present study different LAB strains were evaluated for their ability to antagonize human intestinal pathogens by secretion of antimicrobial compounds, interference with bacterial growth and interference with pathogens during adhesion/invasion process on epithelial cells. The antimicrobial activity of the CFCS of seven LAB strains against five intestinal pathogens was first determined by agar well diffusion method; as reported, two strains, *L. casei* W56 and *L. rhamnosus* W71, showed relative strong antibacterial activity (inhibition zone >15 mm) and all the others strains moderate activity (inhibition zone between 10 and 15 mm) against the tested pathogens. As these cultured broths were neutralized to pH 6.5, the inhibitory activity to pathogenic bacteria is probably due to production of antibacterial molecules rather than to the acidic conditions of the cultural media. Our data are in agreement with several studies referring antimicrobial activity of LAB strains [29, 30]. Whether this is due to production of organic acids (e.g., lactic acid and acetic acid), hydrogen peroxide, bacteriocins or other compounds was not investigated in our study.

Other important properties, linked to beneficial effects of probiotics, are the auto-aggregation and the co-aggregation, defined as the accumulation of bacteria of the same species and the accumulation of bacteria of different species respectively. These properties are fundamental for probiotics since auto-aggregation seems to be correlated with the adherence to epithelial cells [15, 31], a prerequisite for colonization and persistence in the gastrointestinal tract, while co-aggregation represents a barrier to prevent intestinal surface colonization of pathogenic microorganisms [32]. It has been suggested that cellular aggregation could be positive in promoting the colonization of beneficial micro-organisms, as reported for lactobacilli in the gastrointestinal or vaginal tract [33, 34]. In our study, the LAB strains showed relatively higher auto-aggregation compared to intestinal pathogens, suggesting that this property may allow them to survive at sufficiently high number and colonize the gastrointestinal tract. All the LAB strains tested showed co-aggregation abilities with pathogens, with percentages depended on the strain (probiotic and pathogen strains) and time of co-incubation. All the LAB strains showed co-aggregation and aggregation abilities, in particular *B. bifidum* W23 and *L. rhamnosus* W71. Our results are in agreement with Collado et al. [15], who reported a correlation between auto-aggregation and co-aggregation properties. In addition, our results suggest that the ability of LAB to promote co-aggregation with pathogens and to compete for adhesion to the epithelial cell surface is strain-dependent, probably related with the presence of specific molecules in the LAB surface acting either as ligands binding pathogens and/or as adhesins for attachment to epithelial cells [35].

Since the binding to epithelial cells is valuable for probiotic bacteria, we also determined the ability of our LAB strains to adhere to Caco-2 intestinal cells. The adhesion index of the tested strains showed a variability depending on the strain, species and genera; in fact, the most adhesive strain resulted to be *B. bifidum* W23, while, among the *Lactobacillus* spp., the observed adhesion properties differs from species to species. Our data are consistent with studies carried out on the LAB adhesion, showing that this ability was strain-specific and varied within the same species [36–38].

### Interference studies between LAB and intestinal pathogens

To colonize the human gastrointestinal (GI) tract, pathogen bacteria must compete with gut resident microbiota, such as lactic acid bacteria that play crucial roles in maintaining the microbial ecosystem of the GI by preventing colonization and infection of incoming bacterial pathogens [8, 12–14]. It is very important to underline that potential probiotic strains are unique and strains of the same genus and species may have different beneficial effects [14]. In addition, it is assumed that the combinations of specific probiotic strains potentiate the beneficial effects to the host compared to the probiotic strains alone [39]. In this study, after determining the specific properties of individual LAB strains, three strains and their four combinations were selected and tested for their ability to inhibit the invasiveness of *S. enteritidis* ATCC 13076, *L. monocytogenes* ATCC 7644, *E. coli* O157: H7 ATCC 35150, *C. sakazakii* ATCC 29544 and *C. jejuni* ATCC 33291 on Caco-2 monolayers. The LAB selection showed the following specific probiotic properties: *B. bifidum* W23 possessed moderate auto-aggregative and co-aggregative abilities, high antimicrobial activity, and high adhesion index; *L. salivarius* W24 showed moderate auto-aggregative and weak co-aggregative abilities, high antimicrobial ability, and medium adhesion index; while *L. rhamnosus* W71 had high auto-aggregative and co-aggregative abilities, moderate antimicrobial activity, and low adhesion index. Our data demonstrated that each LAB was able to reduce the invasion ability of intestinal pathogens; this is in agreement with other studies reporting the protective effect of lactic acid bacteria against *Salmonella* spp., *L. monocytogenes,* and *C. jejuni* [13, 40–42].

The ability to inhibit the invasion of intestinal pathogens indicates a very high strain-specificity. In fact, *B. bifidum* W23 and *L. rhamnosus* W71 were able to reduce the invasion of the tested pathogens by exclusion as well as by competition, while *L. salivarius* W24 prevalently appeared to operate exclusively via exclusion. Regarding the putative mechanisms of bacterial antagonism, co-aggregation could be one probiotic mechanism of action to prevent the attachment of pathogens to the intestinal surface and avoid its binding to the cellular line [13, 43]. In our case, *B. bifidum* W23 and *L. rhamnosus* W71 both co-aggregated well and reduced the invasion of the intestinal pathogens *L. monocytogenes* ATCC 7644 and *C. jejuni* ATCC 33291. On the contrary, *L. salivarius* W24 did not appear to use co-aggregation as its mechanism of action to reduce the invasion of *L. monocytogenes* ATCC 7644 and *C. jejuni* ATCC 33291. These results support the hypothesis that there are multiple mechanisms by which probiotics exert antagonistic action against intestinal pathogens, and since surface components of LAB are implicated in adhesion, co-aggregation and bacteria–bacteria interactions, these phenomena could be probably interrelated.

According to the hypothesis that a combination of LAB strains may be more effective in vivo than single strains [7, 35], we found increased percentages of invasion inhibition by the four LAB strain combinations compared to those of the individual LAB strains. Particularly interesting is the case of *S. enteritidis* ATCC 13076; in fact, when tested against this pathogen, the individual LAB strains reached low percentages of invasion inhibition (maximum value 57.14% by *L. rhamnosus* W71 in the exclusion test), whilst the LAB combinations achieved a greater cumulative percentage of invasion inhibition up to 82.47%. Similarly, regarding *C. sakazakii* ATCC 29544, the LAB combinations have reached higher percentages of invasion inhibition, even whilst most cases of the single LAB strains have gained good results (up to 82.86% of invasion inhibition). These findings demonstrate that the tested LAB combinations possess probiotic properties, supporting the hypothesis that the use of probiotic combinations, selected for their strain-specific characteristics, may increase the beneficial effects on human health [9, 39].

To our knowledge, this is the first work where LAB strains were tested individually as well as in combination by in vitro interference tests with intestinal pathogens. To verify the ability of different LAB strains, also in their combinations, to inhibit the invasion of pathogenic bacteria appears important for the selection of new probiotic microorganisms. For these reasons, the tested LAB may be potential candidates to develop new probiotic combinations to prevent or treat infections by a specific pathogen.

## Conclusions

LAB strains with good abilities to adhere to epithelial cells could be better suited to colonize the intestine. They can act as a barrier to fight pathogens through different competitive mechanisms, like antimicrobial activity, co-aggregation with pathogens, and adherence. For this, among the tested LAB strains, *B. bifidum* W23, *L. salivarius* W24 and *L. rhamnosus* W71 have the best characteristics showing good antimicrobial activity as well as high interference activity with pathogen invasion. In addition, the LAB combinations, with enhanced antagonizing activity against the tested intestinal pathogens, confirm the importance of specific probiotic combinations to potentiate the beneficial effects to the host (Additional file 1: Figure S1).

## Additional file

**Additional file 1: Figure S1.** Representative images of LAB strains adhesion to Caco-2 cells. (a) *B. Bifidum* W23, (b) *L. salivarius* W24, (c) *L. acidophilus* W37, (d) *L. plantarum* W21.
