# Supplementary material for: Strain-specific probiotic properties of lactic acid bacteria and their interference with human intestinal pathogens invasion
Source: Gut Pathog. 2017 Mar 6;9:12. doi: 10.1186/s13099-017-0162-4 (PMC5338089; doi:10.1186/s13099-017-0162-4)
Supplement: Supplementary file 1 — Additional file 1: Figure S1. Representative images of LAB strains adhesion to Caco-2 cells. (a) B. Bifidum W23, (b) L. salivarius W24, (c) L. acidophilus W37, (d) L. plantarum W21. [file 13099_2017_162_MOESM1_ESM.docx]

**Figure S1**. **Representative images of LAB strains adhesion to Caco-2 cells***.* a) *B. Bifidum* W23, b) *L. salivarius* W24, c) *L. acidophilus* W37, d) *L. plantarum* W21.
